# Tipping phenomena in typical dynamical systems subjected to parameter drift

**DOI:** 10.1038/s41598-019-44863-3

**Published:** 2019-06-17

**Authors:** Bálint Kaszás, Ulrike Feudel, Tamás Tél

**Affiliations:** 1 Institute for Theoretical Physics, Eötvös Loránd University, Pázmány Péter Sétány 1/A, H-1117 Budapest, Hungary; 2 Theoretical Physics/Complex Systems, ICBM, University of Oldenburg, 26129 Oldenburg, Germany; 3 MTA-ELTE Theoretical Physics Research Group, Pázmány Péter Sétány 1/A, H-1117 Budapest, Hungary

**Keywords:** Environmental sciences, Statistical physics, thermodynamics and nonlinear dynamics

## Abstract

Tipping phenomena, i.e. dramatic changes in the possible long-term performance of deterministic systems subjected to parameter drift, are of current interest but have not yet been explored in cases with chaotic internal dynamics. Based on the example of a paradigmatic low-dimensional dissipative system subjected to different scenarios of parameter drifts of non-negligible rates, we show that a number of novel types of tippings can be observed due to the topological complexity underlying general systems. Tippings from and into several coexisting attractors are possible, and one can find fractality-induced tipping, the consequence of the fractality of the scenario-dependent basins of attractions, as well as tipping into a chaotic attractor. Tipping from or through an extended chaotic attractor might lead to random tipping into coexisting regular attractors, and rate-induced tippings appear not abruptly as phase transitions, rather they show up gradually when the rate of the parameter drift is increased. Since chaotic systems of arbitrary time-dependence call for ensemble methods, we argue for a probabilistic approach and propose the use of tipping probabilities as a measure of tipping. We numerically determine these quantities and their parameter dependence for all tipping forms discussed.

## Introduction

In nonlinear systems one can often observe qualitative differences in the asymptotic behavior belonging to different parameter sets. Bifurcations, instabilities, catastrophes can occur^1^. Such phase transition-like phenomena are found even in low-dimensional dynamical systems. There is an increasing interest in the dynamics of systems subjected to a *time-dependent change* of the parameter sets, a phenomenon called parameter drifts, or ramping. Within the realm of such problems, tipping phenomena, i.e. dramatic changes in the asymptotic and transient behaviour, are of special interest, and are found in different branches of sciences^2–4^. We mention four basic examples

- climate dynamics: climate change witnessed in our everyday life as a consequence of changes in the Earth System’s temporal evolution due to, e.g., a slow increase of greenhouse gases^4–6^,
- regime shifts in ecology: implying abrupt, persistent changes in the characteristic behaviour of ecosystems due to changes in internal processes and/or external drivers^7–10^,
- disease spreading in human populations: changes in environmental factors and socio-economic conditions may induce susceptibility to outbreaks^11–13^,
- aging or detuning in engineering problems or dynamical systems when the parameter drift is a result of spontaneous or man-driven processes^14–16^.

Tipping dynamics can best be understood in *multistable systems*^17–19^, where qualitatively different stable asymptotic states coexist for a given set of parameters and/or forcings. Such systems often exhibit bifurcations when a parameter is varied. If a parameter is slowly drifting, bifurcations leading to tipping occur often with a delay depending on the rate of the drift^20–26^. The literature refers to this phenomenon as *bifurcation-induced tipping*^27–29^, which arises when the stable states can be tracked while the parameters are changing. Recently, another type of tipping has been discovered when the rate of parameter drift becomes larger than a critical rate. *Rate-induced tipping*^30^ can take place if tracking fails^28^ or even in situations where no bifurcation is involved^30–34^.

These results, supported by rigorous arguments, are found in systems exhibiting only fixed point attractors (equilibria). Furthermore, tipping is usually considered from the perspective of an individual trajectory starting on one attractor and ending on a different one. The case of periodic attractors has been included only recently^35,36^, and ref.^36^ showed that for periodic attractors partial tipping can occur in the sense that certain phases of the periodic attractor tip and others do not.

Our aim is to investigate what kind of tipping phenomena might occur in typical dynamical systems, also possessing chaotic dynamics, or more generally, systems with topological complexity. Ott and coworkers^37–39^ were the first to study this problem, but only for very slow parameter drifts. We consider the interesting case where no time-scale separation occurs and hence qualitative changes in the dynamics cannot be interpreted as bifurcation-induced tippings. In such cases one cannot hope for analytic results, therefore, we report numerical findings obtained in a paradigmatic model whose behaviour characterizes deterministic nonautonomous low-dimensional dissipative dynamical systems. It was shown in^15,16^ that in systems exhibiting chaotic dynamics, full tracking is impossible, the attractors obtained in the classical bifurcation diagram (which corresponds to an infinitely slow parameter drift) are never similar to the actual attractors in the range of non-negligible rates. For distinction, we will use the term “frozen-in” for systems with fixed parameters. We show that due to the strong internal variability of such complex systems, *tipping probabilities* should be used as measures of tipping. Thus, partial tipping^36^ proves to be the rule rather than the exception. Where chaotic attractors exist, *tipping into chaotic attractors* and *tipping from or through chaotic attractors* can occur, and we find that even initially arbitrarily close trajectories tip into different attractors, resulting in a completely random tipping due to the appearance of riddled-like basins of attraction. In transitions between two qualitatively different regular attractors, *fractality-induced tipping* takes place characterized by a refined dependence on the particular scenario, as a consequence of the ubiquitous presence of fractal basin boundaries. A less apparent effect of underlying transient chaos is *transient-reduced tipping* characterized by a reduction of the probabilities when tipping processes are studied on finite observation times, a frequent situation in research. We find rate-induced tippings appearing in form in which tipping probabilities increase gradually from zero when the rate is increased. This is in contrast to the sharp transitions recorded on the level of individual trajectories in systems lacking topological complexity^30^. We conclude that the existence of chaotic motion leads to a multitude of new tipping phenomena induced by topological complexity.

## Bifurcation Diagram of the Frozen-in System and Parameter Drift Scenarios

**Figure 1:**
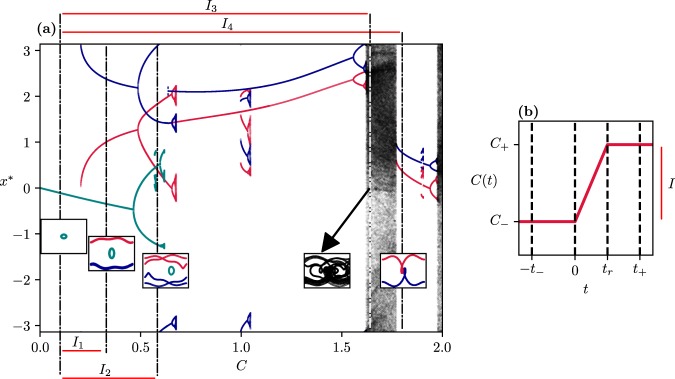
(**a**) Bifurcation diagram of a typical nonlinear system with frozen-in control parameter *C*: the *x*-coordinates of the long-term attractors are shown on a stroboscopic map. (**b**) Schematic diagram of the parameter drift scenario used in the paper. The scenario covers a control parameter interval *I* = [*C*_−_, *C*_+_] corresponding to a control parameter change Δ*C* = *C*_+_ − *C*_−_. The limiting *C* values of the scenarios are marked by vertical dashed-dotted lines in (**a**). The continuous-time attractors belonging to these values are given in the insets. The control parameter intervals *I*_*n*_, *n* = 1, …, 4 investigated in the main text are marked by line segments along the frame of the bifurcation diagram.

When investigating systems with a parameter drift, it is worth looking at first the long-term dynamics depending on the parameter without the intrinsic drift. This results in a classical bifurcation diagram for the frozen-in system. Here we are particularly interested in systems which are periodically forced. This forcing with a certain period *T* would correspond to the seasonal variability of the climate or of certain environmental drivers over the period of one year, like e.g. temperature for a natural or societal system, or periodic energy input into a mechanical system. The strength of the forcing would be the main control parameter *C* to be varied. A typical bifurcation diagram for such a system is presented in Fig. 1a. It shows various stable long-term dynamics, usually called attractors (x^*^ represents one of the state space variables on them), which in general could be regular or chaotic, depending on the fixed control parameter *C*. When the system possesses a periodic or seasonal forcing it is convenient to plot the state of the system as a stroboscopic map, or Poincaré section, i.e. always at the same time instant in each period of the forcing. This corresponds to measuring the time in units of the period *T* of the forcing. For the paradigmatic system of a periodically forced pendulum studied here as an example (cf. Supplemental Discussion I for a detailed description) only periodic and chaotic attractors exist. While chaotic dynamics corresponds to the black areas filled irregularly with points in Fig. 1a., the different periodic motions appear as curves. Out of these, swinging motion about the equilibrium of a resting pendulum (cyan) as well as clockwise and counter clockwise rotations (red and blue) are discerned. The latter are born (along with their unstable counterparts) in a saddle-node bifurcation^1^ at *C*_*b*_ = 0.2, and exist throughout the *C*-range shown except for chaotic regimes. Small, multi-piece attractors are also visible in short parameter intervals.

Insets show the continuous-time attractors in state space (*x*, *y*) associated with a few selected values of the control parameter *C*. These will serve as limiting values of the parameter drift scenarios. The stroboscopic view of these attractors presented in the bifurcation diagram is obtained by cutting through these curves with a line. The strikingly different geometrical appearance of these attractors implies qualitatively different long-term dynamics in the frozen-in system.

The bifurcation diagram suggests a rather limited presence of chaos in the dynamics. However, we should emphasize that bifurcation diagrams are known to not represent an important aspect of the dynamics, namely transients. In typical nonlinear systems, transients can be chaotic, and their existence due to an underlying unstable chaotic set, called a chaotic saddle^40–42^, might extend over a considerable range of the control parameter.

Let us now consider the scenario of a parameter drift, i.e. a case when the control parameter becomes time-dependent following a known function *C*(*t*). One possibility would be that the control parameter starts at some initial value *C*_−_, changes initially rather weakly, then undergoes a rapid change in some time interval before ending at *C*_+_ > *C*_−_^30^. We consider here a simpler case, a piece-wise linear parameter drift involving a ramp, i.e., a linear increase of size Δ*C* = *C*_+_ − *C*_−_, starting always at time *t* = 0 and ending at the ramping time *t* = *t*_*r*_. The rate *r* of the parameter drift in the time interval 0 < *t* < *t*_*r*_ is thus given by

1 $$r=\frac{{\rm{\Delta }}C}{{t}_{r}}.$$

Such ramping scenarios are rather common in the climate literature (see e.g.^43,44^), and have been used to study critical transitions and regime shifts^7,8^, also describing parameter detuning in physical and dynamical systems (see e.g.^37–39^).

Typically, our investigations start at some initial time *t* = *t*_−_ ≤ 0, with the initial *C*_−_ value on a plateau and end at time *t* = *t*_+_ > *t*_*r*_ on a higher plateau of value *C*_+_ (cf. Fig. 1b for a schematic diagram). The parameter intervals *I* used in the paper are marked by line segments below and above the frame of Fig. 1a.

The full scenario is determined by 5 parameters: *C*_−_, *C*_+_, *t*_−_, *t*_*r*_, *t*_+_. The infinite past and infinite future is approximated by the conditions |*t*_−_|, *t*_+_ ≫ *t*_*r*_. Thanks to the plateaus, where the dynamics takes place with frozen-in parameters, the asymptotic attractors at *C*_+_ and *C*_−_ can be determined as usual. In order to ensure a time scale that is comparable to those of the frozen-in dynamics (which are in the range[1, 10]), we will focus on the range *t*_*r*_ < 40.

The main effect of the parameter drift is that states initially close to the attractor at *C*_−_ (which would indeed converge to this attractor without ramping (Δ*C* = 0)) might in fact converge to a completely different attractor (exemplified by the insets in Fig. 1a). We refer to this qualitative change in the asymptotic behavior as a tipping transition.

In order to find a quantitative measure for the probability of possible tipping transitions, we are interested in the *scenario-dependent* changes of the basins of different attractors. The basin of attraction of a particular attractor is the set of initial conditions, distributed uniformly over the state space, which converges to it. Here, we will determine the basins of the attractors at *C*_+_, *t*_+_ for initial conditions taken at an initial time *t*_−_ and control parameter value *C*_−_. The result will depend not only on the interval *I* of the parameter drift, but also on the other, temporal parameters of the scenario. It is worth noting that the special case of ramping time *t*_*r*_ = 0, *t*_−_ = 0, corresponds, for large *t*_+_, to the basin of attraction for the frozen-in system with *C*(*t*) = *C*_+_.

## Tipping Transitions in Typical Systems, Tipping Probabilities

In systems possessing chaotic attractors, or more generally, systems with topological complexity, *individual trajectories are not representative*, due to the presence of unpredictability^41^. Therefore, we follow an *ensemble approach in the context of tipping*, i.e., we start with a large number of trajectories distributed over the full state space and determine which of them converge to the given attractors. Since typically different attractors *coexist* at *C*_+_, only a certain portion of the ensemble will approach any given attractor. This implies that partial tipping takes place in the terminology of  ^36^.

Let us assume that at *C*_−_ there exists only a single attractor, such that the whole ensemble of initial conditions would converge to it in the frozen-in system. It might happen that this attractor continues to exist up to *C*_+_. If so, along with the continuation of the original one, there might exist one or even more additional attractors at *C*_+_. We now ask the question, which initial conditions of the ensemble, spread over the state space at *C*_−_ at time *t* = *t*_−_, would end up on one of the additional attractors at *C*_+_, instead of the continuation of the one existing at *C*_−_. In other words, we determine the basins of attraction of the attractors at *C*_+_, while taking the drifting process into account. Since they depend on all the parameters of the scenario, we call them *scenario-dependent basins*. We say a trajectory in the ensemble tips, when it converges to an attractor at *C*_+_ different from the one it would converge to at *C*_−_. Since not all trajectories will tip in general, we cannot expect sharp switchings between attractors. Instead of the existence of sharp tipping points, one finds gradual transitions between attractors in the form of partial tipping^36^.

As a quantitative measure of the transition we propose the use of *tipping probabilities*. In noisy systems this concept has already turned out to be useful^31^. The novelty of our approach lies in applying tipping probabilities to fully deterministic systems. This quantity is based on the scenario-dependent basins of attraction mentioned above. The tipping probability ${P}_{{A}_{1},{A}_{2}}$ from attractor *A*_1_ to *A*_2_ is defined as the proportion of the part of the area of the basin of attraction of *A*_1_ at *C*_−_ that is mapped to *A*_2_ at *C*_+_ by the end of the scenario at time *t*_+_. This formulation is valid even if several attractors coexist at *C*_−_ (cf. Section Methods). The quantity ${P}_{{A}_{1},{A}_{1}}$ expressing the chance that the system will end up on the continuation of the same attractor it started from, can be called the *tracking probability*. Naturally ${\sum }_{i}{P}_{{A}_{1},{A}_{i}}=1$ holds, where *i* counts the attractors existing at *C*_+_. The tipping probabilities depend of course on all parameters of the scenario (*t*_−_, *t*_+_, *t*_*r*_, *C*_−_, *C*_+_).

## Tippings by Drifting Through a Saddle-Node Bifurcation

**Figure 2:**
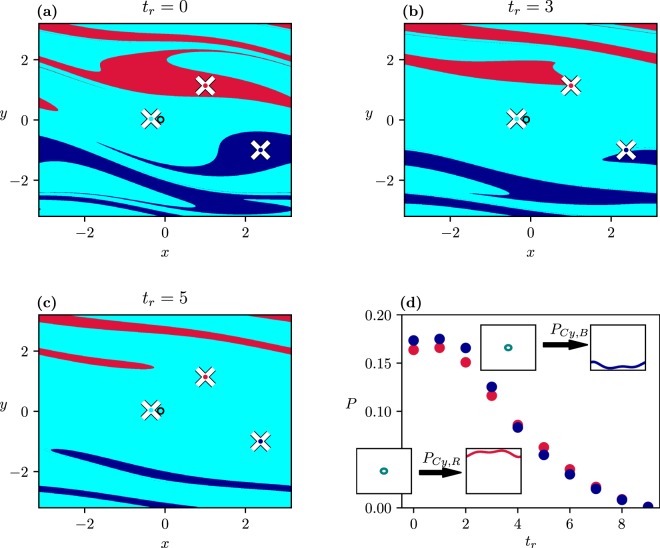
Scenario-dependent basins of the three attractors (colored dots within white crosses) belonging to *C*_+_ = 0.33 with scenarios starting at *t*_−_ = 0, *C*_−_ = 0.1 and ending at *C*_+_ = 0.33 (*I*_1_) by *t*_+_ = 40 for different ramping times: *t*_*r*_ = 0 (**a**), 3 (**b**), and 5 (**c**). The cyan attractor belonging to *C*_−_ is marked by a black circle in the cyan background. (**d**) Dependence of the tipping probabilities *P*_*Cy*,*B*_ and *P*_*Cy*,*R*_ on *t*_*r*_ with a visual illustration of the switch between attractors due to the tipping transitions (insets). The dots representing the tipping probability values are coloured, here and in the following, with the colour of the attractor in the bifurcation diagram of Fig. 1a into which tipping occurs.

As a first case, let us take an interval [*C*_−_, *C*_+_] = [0.1,0.33] (marked by *I*_1_ in Fig. 1a) which contains the saddle-node bifurcation occurring at *C*_*b*_ = 0.2 for both the rotating attractors (in the course of these bifurcations^1^ a stable-unstable pair of periodic solution emerges when passing *C*_*b*_ from below). Figure 2 shows how the scenario-dependent basin structures change with the ramping time when the scenario starts with the ramp: *t*_−_ = 0. Figure 2a belongs to *t*_*r*_ = 0, i.e. to the frozen-in case with *C*_+_. The periodic attractors (appearing as points within white crosses) lie in the middle of the coloured basins, as expected. With increasing *t*_*r*_ the basins deform, Fig. 2b,c, and the rotating (red and blue) attractors move out of their ‘own’ basins of attraction. Note that due to the parameter drift all attractors move in state space and are found in a different location at *t*_+_ than at *t*_−_, while their basins characterize the situation at *t*_−_ = 0. The basin boundaries are smooth curves here.

In Fig. 2d we plot the tipping probability, *P*_*Cy*,*B*_ and *P*_*Cy*,*R*_, i.e. the probability of tipping from the cyan attractor *Cy* of swinging to one of the rotating red or blue (*R*, *B*) attractors. The insets illustrate the qualitative change of the attractors during the transition. Note that at *C*_−_ = 0.1 the basin of attraction of the cyan attractor *Cy* contains the full state space.

These tipping probabilities vanish at *t*_*r*_ = 9, i.e. for slow rates. We can thus conclude that the tipping of a trajectory to an attractor differing from the initial one is rate-induced. Note that rate-induced tipping is related in the literature to a critical rate beyond which tipping happens^27,28^. Since our transition to vanishing tipping probabilities is smooth, such a critical rate cannot be identified. This asymptotic vanishing seems to characterize all the rate-induced tippings occurring in typical systems. Note that the quantity ${P}_{Cy,Cy}=1-{P}_{Cy,B}-{P}_{Cy,R}$, the tracking probability of the cyan attractor (not shown) would be practically unity for *t*_*r*_ > 9.

The traditional approach to tipping^30^ concentrates on trajectories starting on a single attractor at a time −∞, and asks if such a trajectory can end, at a time +∞, on an attractor differing from the continuation of the initial one existing at the end of the scenario. In the ensemble view, this corresponds to choosing a small area around the attractor at *C*_−_ or choosing a large area if the scenario starts at *t*_−_ < 0, |*t*_−_| ≫ *t*_*r*_, which will shrink anyhow to a small one, due to dissipation, by *t* = 0. It is seen in Fig. 2 that a small enough region can always be chosen about the initial attractor (black circle) such that every trajectory inside is colored cyan, i.e., no traditional rate-induced tipping exists in this system.

**Figure 3:**
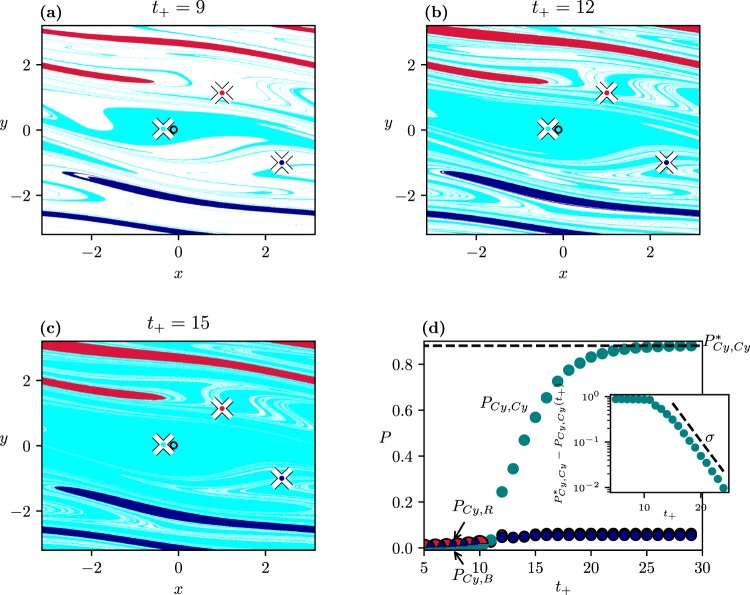
Basins of attraction with the scenarios of Fig. 2 observed at *t*_+_ = 9(*a*), 12(*b*), 15(*c*), at a fixed *t*_*r*_ = 5. (**d**) Dependence of the tipping probability (into any of the 3 attractors at *C*_+_) on *t*_+_. The inset indicates the function ${P}_{Cy,Cy}^{\ast }-{P}_{Cy,Cy}$ vs *t*_+_ on a semi logarithmic scale of asymptotic slope *σ* = 0.385 ± 0.005.

Additionally, we find a new phenomenon if the investigation does not end at “infinity”, but rather at finite times. As Fig. 3a–c shows, large white regions appear in the state space marking initial points which have not yet converged to any of the attractors at *C*_+_. Moreover, the boundary of the white region is interwoven with the coloured ones in a fractal-like fashion. This should be interpreted as a consequence of the existence of *transient chaos*^42^ both in the frozen-in and in the ramped system. A portion of the trajectories converges then to a chaotic saddle, coexisting with the periodic attractors. These are trajectories which start in the vicinity of the stable manifold of the saddle, and then spend some time around the saddle.

The area of the white region is of course shrinking with *t*_+_. Figure 3d shows the dependence of the tipping probability on *t*_+_(≥*t*_*r*_). Transients are so long that for small values of *t*_+_ − *t*_*r*_ only a small portion of all the trajectories reaches any of the attractors, and this portion happens to be practically the same for all attractors. In harmony with the starting dominance of the cyan dots in Fig. 3b, the tracking probability *P*_*Cy*,*Cy*_ starts increasing at about *t*_+_ = 12, and reaches a high plateau at ${P}_{Cy,Cy}^{\ast }$ later. By contrast, the tipping probability into the rotating attractors increases only slightly, remains practically the same and saturates at a low value corresponding to that of Fig. 2d. The inset displays the tracking probability difference: ${P}_{Cy,Cy}^{\ast }-{P}_{Cy,Cy}$ vs *t*_+_, which exhibits a long-term exponential decay.

**Figure 4:**
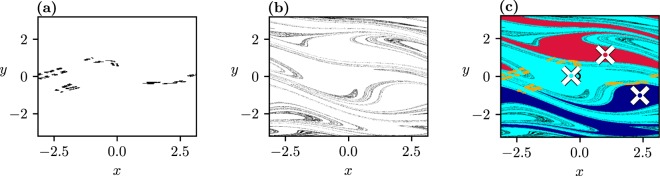
(**a**) Chaotic saddle existing in the frozen-in system at *C* = 0.33. (**b**) Stable manifold of the saddle. Both objects are determined by the sprinkler method as described in^41,42^. (**c**) The saddle (yellow dots) and its stable manifold laid over the basins of attraction in the frozen-in system, with *C*_+_ (Fig. 2a).

To illustrate the relevance of transient chaos, we show in Fig. 4 the frozen-in chaotic saddle at *C*_+_, its stable manifold, and the relation of these to the basin patterns found at this *C*_+_. We see that transient chaos is restricted entirely to the cyan basin, as the saddle does not intersect the boundary. Since this appears to be the case for any *C* in interval *I*_1_, this explains the smooth, nonfractal nature of the boundaries of Fig. 2, and shows that transient chaos appears here in a somewhat hidden form but influences the basin structures in the ramped system when the observation time is short.

From dynamical systems theory it is known that any trajectory starting close to a chaotic saddle will escape its neighbourhood after some time^42^. The escape rate *κ* governing the decay of points *N*(*t*) remaining near the frozen-in saddle up to time *t*, according to the rule $N(t)\sim \exp (-\kappa t)$^42^, is found to be *κ* = 0.365 ± 0.001. The strong relation between transient chaos and the number of points not yet converged in the ramped system is reflected by the temporal decay of ${P}_{Cy,Cy}^{\ast }-{P}_{Cy,Cy}$. This quantity also decays exponentially, as exp(−*σt*) with *σ* = 0.385. This *σ* can be interpreted as the escape rate governing the whole ramped process. The fact that the *σ* and *κ* values are close suggests that the decay dynamics of the white area in the ramped system is dominated by the escape rate of the saddle existing on the *C*_+_ plateau.

The strong *t*_+_-dependence of the fractal-like boundary between cyan and white is found to characterize all cases with *C*_+_ > *C*_*b*_, since transient chaos appears to be present for almost all *C* values in this range. Such a *t*_+_-dependence cannot be found in systems without underlying topological chaos, which is the case for the current literature, see e.g.^30^.

In summary, the presence of transient chaos is reflected by the appearance of fractality in the scenario-dependent basin boundaries for finite observation times, in spite of the smooth boundaries characterizing cases with sufficiently long observation times. Meanwhile, the tipping probabilities for small *t*_+_ become smaller, a phenomenon we call *transient-reduced tipping*. In reality, we often have to restrict ourselves to observation times which are not very long. One should keep in mind that tipping processes in such cases might differ from the ones characterized by long observations. The time period over which reduction is present is a few times 1/*σ*, the characteristic time of the transients’ decay in the ramped process:

2 $${t}_{+}-{t}_{r}=\alpha \frac{1}{\sigma },$$

where *α* is a number on the order of unity. Since *σ* might be arbitrarily small in transient chaos^15,42^, the time of reduction can be surprisingly long.

## Tippings Ending at Higher Order Periodic Attractors

**Figure 5:**
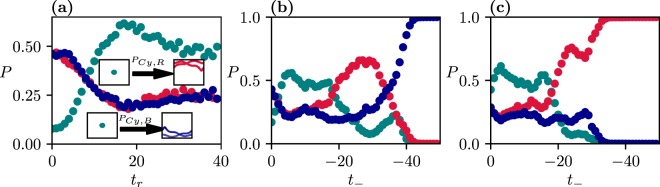
(**a**) Dependence of the tipping probabilities on *t*_*r*_ for *t*_−_ = 0, *C*_−_ = 0.1, *C*_+_ = 0.5835 (*I*_2_) and *t*_+_ = 100 (in order to avoid transient-reduced tipping discussed in the previous section), with a visual illustration of the change in the character of the attractors due to the tipping transitions (insets). (**b**) and (**c**) Dependence of the tipping probabilities on the starting time *t*_−_ with *t*_*r*_ = 5 in (**b**) and *t*_*r*_ = 10 in (**c**). Since the tipping probabilities are heavily dependent on both *t*_*r*_ and *t*_−_, we get a more complete view by showing this bivariate function in Supplemental Discussion III.

Let us now consider a scenario that drifts through several bifurcations. In the example of this section, we start with *C*_−_ = 0.1 and end at *C*_+_ = 0.5835 (interval *I*_2_ in Fig. 1a) where the frozen-in system has period-two attractors, possessing basins of attraction with a fractal boundary, indicating that chaotic transients are much more prominent. This applies also to the scenario-dependent basins (cf. Supplementary Discussion IIA). The tipping probabilities are given in Fig. 5a. In contrast with the scenario of the previous section, the probabilities do not decay quickly, but saturate up to *t*_*r*_ ≈ 40. This property has to do with the emergence of complex structures in the scenario-dependent basins. The plateaus last up to about 80 time units, then *P*_*Cy*,*R*_ and *P*_*Cy*,*B*_ start decaying, and these probabilities approach practically zero at *t*_*r*_ = 120. The quasistatic limit of perfect tracking is thus reached for *t*_*r*_ > 120.

**Figure 6:**
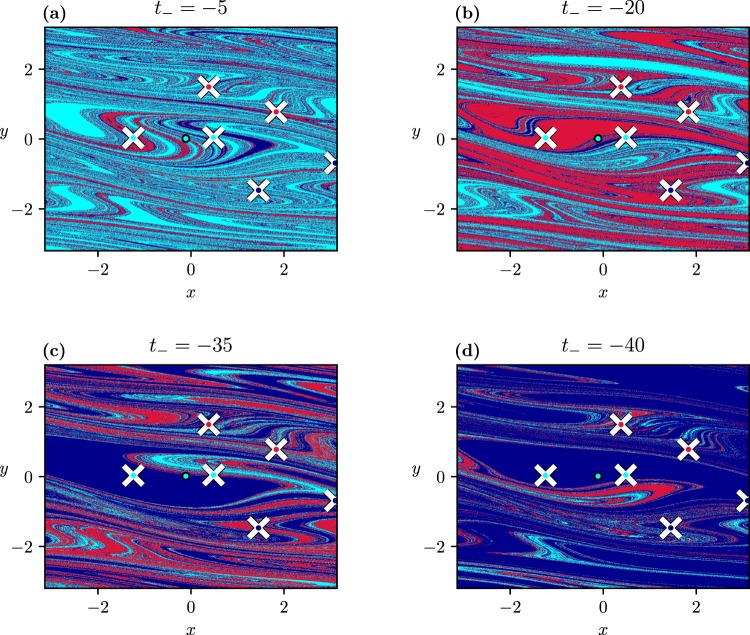
The dependence of the scenario-dependent basins of attraction on *t*_−_ with scenarios starting at *C*_−_ = 0.1 ending at *C*_+_ = 0.5835, *t*_+_ = 100 (*I*_2_), with *t*_*r*_ = 5. For each scenario, *t*_−_ is indicated above the panels and the tipping probabilities can be read off from Fig. 5b. The basin belonging to *t*_−_ = 0 is given in S. Fig. 3b.

A surprising new phenomenon occurs when investigating the dependence of the basin structure on the starting time *t*_−_ < 0 of the scenario. As Fig. 6 shows, there is a strong change in the dominant attractor back to *t*_−_ = −40, after which blue dominates. This seems to contradict the naive expectation according to which going further and further into the past on the *C*_−_ plateau should correspond to leaving more and more time for the ensemble to converge to the single (cyan) attractor existing there, from which then no escape is possible.

The strong *t*_−_-dependence up to −40, and the saturation afterwards, is clearly visible also in the tipping probabilities shown in Fig. 5b,c. For |*t*_−_| sufficiently large, all of the considered trajectories converge to the same attractor, which represents a complete tipping. Interestingly, the particular attractor chosen by the scenario depends on *t*_*r*_ as a comparison of Fig. 5b,c indicates. Thus, the attractors describing rotations behave in a strongly different way due to the parameter drift, although they are dynamically equivalent in the frozen-in system (see Supplemental Discussion I).

**Figure 7:**
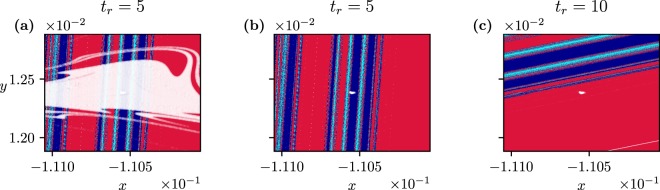
Patches (drawn in white) of the ensembles initiated on the full state space at *t*_−_ = −29 (*t*_−_ = −45) in (**a**) [(**b**),(**c**)] displayed at *t* = 0. The patches are laid over the scenario-dependent basins of attraction, for the scenarios *t*_−_ = 0, *t*_+_ = 100, *C*_−_ = 0.1, *C*_+_ = 0.5835 (*I*_2_) with *t*_*r*_ given above the panels. Note the strong magnification of the basins about the cyan attractor at *C*_−_.

To find an explanation, we follow an ensemble of trajectories, distributed initially uniformly over the full state space, as it evolves in time, from *t* = *t*_−_ < 0 to *t* = 0. All these points converge of course towards the single (cyan) attractor existing for *C*_−_. Because *t*_−_ is finite, the convergence is yet incomplete but the patch formed by the ensemble is smaller for larger |*t*_−_|. When reaching the time instant *t* = 0 (when *C* starts to change along the ramp), we overlay the patch with the scenario-dependent basins of attraction obtained for a scenario starting at *t*_−_ = 0 and with all the other parameters unchanged. A sufficiently contracted patch can be considered to have converged to the (cyan) attractor at *C*_−_ but its precise small-scale structure turns out to be relevant from the point of view of tipping. The intersection of the patch with any of the scenario-dependent basins provides those trajectories that will tip to the corresponding attractor. In this context, the surprising, almost certain tipping for |*t*_−_| > 40 can be understood as follows. Figure 7 shows the patch placed on the corresponding scenario-dependent basins of attraction, for two different *t*_−_-s. We see that the basins’ small-scale structure is the cause of the change in the character of tipping. The basins are made up of unicolored regions separated by fractal boundaries. Because of this, since at the time instant when the parameter drift starts (*t* = 0), the small patch of the initial ensemble might overlap with several colors (Fig. 7a), but for a sufficiently large |*t*_−_| it fully falls into one of the basins (Fig. 7b). This means, that one of the frozen-in attractors is selected by the scenario, and trajectories tip to it with probability 1. The exact outcome of the tipping of course depends on *t*_*r*_. This can be thought of as the patch remaining unchanged, but the underlying basin structure changing, depending heavily on *t*_*r*_, as illustrated by comparing Fig. 7b,c.

We call the strong variability in the tipping probabilities, before the asymptotic behaviour sets in, *fractality-induced tipping*, as it is a consequence of the fine fractal structure of the scenario-dependent basin boundaries.

It is also of interest to investigate scenarios in which the initial control parameter *C*_−_ allows more than one coexisting attractors in the frozen-in system. Then, it should be taken into account that the basin of attraction at *C*_−_ of an attractor *A*_1_ is only a portion of the full state space, and the tipping probability from *A*_1_ to any other attractors should be determined from trajectories starting from this basin. The details are given in Supplementary Discussion IIB.

## Tippings when Extended Chaotic Attractors are Involved

When the scenario ends at a *C*_+_ belonging to a single extended chaotic attractor (interval *I*_3_ in Fig. 1a), a global type of tipping occurs. Here the end state is not a limit cycle represented by a few points in the stroboscopic map, but rather an extended chaotic attractor of fractal structure, possible on *intervals* in the bifurcation diagram of Fig. 1a, or a coil as in the 4th inset there. We call this transition *tipping into a chaotic attractor*. In this case, all initial conditions lead to an unpredictable motion. It is worth emphasizing that these features hold for *all scenarios* starting at any *C*_−_ > 0 with any *t*_−_, *t*_*r*_, provided *t*_+_ is sufficiently large. Since the chaotic attractor at *C*_+_ is a single globally stable attractor for our system, the tipping probability into this attractor is *unity* starting from *any* attractor that may exist at *C*_−_.

**Figure 8:**
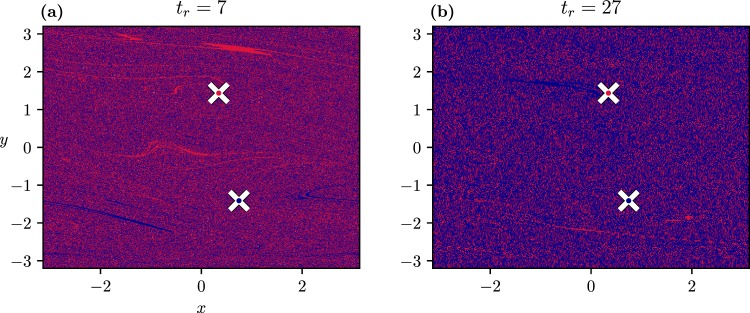
Scenario-dependent basins of attraction for a parameter drift scenario ending at *C*_+_ = 1.8 (after an extended chaotic regime, interval *I*_4_ in Fig. 1a) with *t*_−_ = 0, *t*_+_ = 100. (**a**) *C*_−_ = 0.1, *t*_*r*_ = 7, and (**b**) *C*_−_ = 0.1, *t*_*r*_ = 27.

Next, let us consider a final parameter value *C*_+_ lying beyond the extended chaotic attractor where again only the two rotating motions are attracting. Choosing for simplicity a low value of *C*_−_ = 0.1 where a single attractor exists (see interval *I*_4_ in Fig. 1a), one finds the basin structure shown in Fig. 8a,b. We see that in the near vicinity of any colour there is a neighbouring point of the other colour: the basins are riddled-like^42,45,46^.

The dynamics on the extended chaotic attractor is so strongly mixing that in *any* scenario passing through such an attractor the tipping probabilities from the initial attractor to both of the rotating periodic ones are found to be practically 1/2. Figure 8a,b illustrates that the pattern hardly depends on *t*_*r*_. Predictability of individual trajectories is completely lost and one can speak of *random tippings* in such cases. The fact that the tipping probabilities are independent of the choice of *C*_−_ imply an important special case. When *C*_−_ (<*C*_+_) belongs to an extended chaotic attractor, the probabilities remain 1/2 and the process is fully unpredictable and random. We call such random tipping transitions *tipping from or through a chaotic attractor*.

Scenarios ending or starting on small-size chaotic attractors and a comparison of different *I* intervals of the same length are investigated in Supplementary Discussions IIC and IID. In Supplementary Discussion IV we study the role of snapshot attractors (see Methods) in the tipping transitions. We show that after arriving at the plateau of *C*_+_, the snapshot attractor starts to develop peaks about the usual periodic attractors. The weight of these peaks determines the tipping probabilities, and they, just like the time evolution of the snapshot attractor, depend on the entire scenario.

## Conclusions

In this paper we have investigated tipping phenomena due to parameter drifts in typical dynamical systems exhibiting permanent or transient chaotic motion (due to topological complexity) in the frozen-in case. Because the rates of the parameter drifts are on the same order of magnitudes as the time scales of the internal dynamics, no bifurcation-induced tipping can occur.

The chaotic nature of the dynamics requires the consideration of ensembles of trajectories instead of single trajectories. Surprisingly, in spite of the deterministic nature of the problem, we find that transitions take place with a certain likelihood. Associated *tipping probabilities* are shown to be a powerful quantitative measure to characterize the tipping process. The tipping probabilities depend smoothly (but not necessarily monotonously) on the drift scenario. In contrast to traditional tippings related to a critical rate for the parameter drift, we find no sharp transitions but a rather gradual vanishing of tipping probabilities which we still consider as a type of rate-induced tipping.

We argue, that *scenario-dependent basins of attraction* containing all initial conditions converging to a particular attractor in the ramped system are the key to understanding the different tipping transitions. We explored the following novel types of tipping (for more details, see Supplementary Information).

- (i): *Tipping into a chaotic attractor* occurs when the system starts from a periodic attractor but ends up on a chaotic one, allowing a plethora of permitted states.
- (ii): *Tipping from or through a chaotic attractor* is closely related to losing the predictability of which trajectories tip and which do not. As a result of the unpredictability of the dynamics on chaotic attractors, tipping is random from the perspective of the individual trajectory.
- (iii): *Fractality induced tipping* happens when the scenario-dependent basins exhibit fractal structures. These lead to non-monotonicities of tipping probabilites.
- (iv): *Transient-reduced tipping* is found when the observation time of a tipping process is short, and observations of natural and technical processes always face this problem, tipping probabilities will be underestimated. This effect is found to be strongest if the transients are chaotic and long-lived.

Most of the novel phenomena found are due to the topological complexity underlying typical dynamical systems. Therefore we expect these to appear in all four disciplines mentioned in the introduction: climate science, ecology, disease spreading, and engineering applications. Since chaotic transients are known to possibly have very long lifetimes in high-dimensional systems as e.g. spatio-temporal systems^42,47^ and/or systems possessing a large multitude of coexisting attractors^48^, the tipping phenomena described here might play a relevant role in such cases, too. Finally, we emphasize that the essence of these novel types of tipping phenomena can best be understood in terms of snapshot attractors associated with the drifting dynamics determined by the scenario in question.

## Methods

### Snapshot attractors

The traditional view on tipping is based on cases where the system dwells on a single (regular) attractor and ends on another attractor. Tipping in this view means that there exists in the ramped system at least one trajectory that starts on one attractor and ends outside the continuation of this attractor at the end of the parameter drift. In typical systems, however, one has to allow for a broader picture, in which snapshot attractors play an important role.

The literature on the investigation of chaotic-like systems with parameter drifts indicates the need of the use of *ensemble* approaches^49–60^ because individual trajectories prove to be not representative. This view has also been applied to many-degrees-of-freedom systems^44,61–67^, and to experiments^68,69^. The unifying concept beyond these approaches is that of snapshot attractors^70^ (also called pullback attractors in the mathematics and climate-related literature^57,71–74^). Loosely speaking, a snapshot attractor is an object belonging to a given time instant that is traced out by an *ensemble* of trajectories initialized in some chosen region of the state space in the past, with all of the ensemble members governed by the same equation of motion. Due to dissipation, the ensemble rapidly forgets the initial conditions, and the snapshot attractor is reached, from a practical point of view, after a typically short period of time. The case where two or more snapshot attractors coexist (multistability of snapshot attractors) is of special interest. The investigations of Pierini *et al*.^63^ provide the first example for the coexistence of a chaotic and a periodic snapshot attractor. In this problem, however, the parameter scenario is that of an irregular temporal oscillation about a mean, and the phenomenon of tipping has not been investigated.

### Tipping probabilities

The state space of systems is typically unbounded, but in practice, one studies a smaller region of it. An appropriate overview of the dynamics is expected if this chosen region contains all relevant attractors. In order to obtain a formula for the tipping probability, one investigates the basin of attraction restricted to this considered region, what we call a relative basin.

In order to determine the tipping probability ${P}_{{A}_{1},{A}_{2}}$, first one determines the *scenario-dependent* relative basin of attraction, ${B}_{2}^{{\rm{sc}}}({t}_{-},{t}_{+})$, of *A*_2_ existing *by the end of the scenario* (at control parameter *C*_+_) under the *ramped* dynamics. Next we take its intersection with the relative basin of attraction *B*_1_ of attractor *A*_1_ *frozen-in* at the parameter *C*_−_ belonging to the initial time *t*_−_. A general formula for this tipping probability can then be given as

3 $${P}_{{A}_{1},{A}_{2}}=\frac{|{B}_{2}^{{\rm{sc}}}({t}_{-},{t}_{+})\cap {B}_{1}({t}_{-})|}{|{B}_{1}({t}_{-})|}.$$

The area of the intersection then needs to be compared to the area of *B*_1_, as Eq. (3) shows. Taking the intersection of the basins ${B}_{2}^{{\rm{sc}}}({t}_{-},{t}_{+})\cap {B}_{1}({t}_{-})$ in the numerator expresses the fact that those trajectories are said to trip which emanate from the basin of *A*_1_ and end up on *A*_2_ as a result of the parameter drift. The applicability of such probabilistic concepts to deterministic systems is a consequence of the disorder or irregularity of chaotic dynamics resembling noisy dynamics to a certain extent.

The authors of  ^75^ coined the concept of survivability as the fraction of initial conditions giving rise to evolutions that stay within a desirable region of the state space all the time. This concept is similar but different from that of the tracking probability since we only compare initial and final states. The tracking probability ${P}_{{A}_{1},{A}_{1}}$ corresponds to end-point tracking in the terminology of Ashwin and coworkers^30^.

Finally we mention that all relative basins of attraction and the corresponding tipping probabilities are computed from stroboscopic sections determined by the phase of the periodic component of the driving. We have chosen *t* = 0 mod *T* as the phase for all computations. Choosing another phase would lead to slightly different structures of the basins of attraction but would not change the results qualitatively.

### Numerical methods

We obtain the numerical results by solving the system of ordinary differential equations detailed in Supplementary Discussion I, by a Runge-Kutta scheme with adaptive stepsize-control. At *t*_−_, we distribute *N* = 10^6^ points uniformly in the dimensionless state space region (−*π*, *π*) × (−3.2, 3.2) (the considered region of the state space). Since all possible attractors for all the considered scenarios are located within this box, we have referred to it in the main text as the “full state space”.

The scenario-dependent basin of attraction ${B}_{2}^{{\rm{sc}}}({t}_{-},{t}_{+})$ of *A*_2_ is determined by integrating all the trajectories uniformly spread over the “full state space” running from *t*_−_ forward to *t*_+_. At the end of this simulation we mark, in the considered region, those trajectories which have converged to *A*_2_ by *t*_+_. The criterion for convergence is that the trajectory gets sufficiently close to the attractor. This is checked, in each period, by computing the distance in state space between the trajectory and points on the attractor with the threshold for convergence chosen to be 0.05.

## Supplementary information

Supplementary Information

## Data Availability

The algorithms used to generate the data may be accessed from the webpage https://github.com/balintkaszas/DSTipping.
