# Supplementary material for: Tipping phenomena in typical dynamical systems subjected to parameter drift
Source: Sci Rep. 2019 Jun 17;9:8654. doi: 10.1038/s41598-019-44863-3 (PMC6572843; doi:10.1038/s41598-019-44863-3)
Supplement: Supplementary file 1 — Supplementary Information [file 41598_2019_44863_MOESM1_ESM.pdf]

Tipping phenomena in typical dynamical systems subjected to parameter drift: Supplementary Information

Bálint Kaszás,

Institute for Theoretical Physics, Eötvös Loránd University,

Pázmány Péter Sétány 1/A, H-1117 Budapest, Hungary

Ulrike Feudel,

Theoretical Physics/Complex Systems, ICBM,

University of Oldenburg, 26129 Oldenburg, Germany

Tamás Tél,

Institute for Theoretical Physics, Eötvös Loránd University,

Pázmány Péter Sétány 1/A, H-1117 Budapest, Hungary, and

MTA-ELTE Theoretical Physics Research Group,

Pázmány Péter Sétány 1/A, H-1117 Budapest Hungary

SUPPLEMENTARY DISCUSSION I: THE MODEL

We consider the paradigmatic example of a pendulum with a periodically forced suspension point, where the amplitude C of this forcing changes in time. The dimensionless equation of motion for the angle x, taken with respect to the vertical, of this driven mathematical pendulum reads as [35]:

$$\dot{x} = y, \qquad \dot{y} = -\gamma^2 \sin(x) - 2\beta y + C(t) \cos(x) \cos(t). \tag{S1}$$

The dimensionless driving period is $T = 2\pi$, chosen as the time unit throughout the paper. Parameter γ represents the dimensionless frequency of the pendulum's small amplitude swingings without driving and dissipation, and β is a friction constant. In our numerical study we fix the values of $\gamma = 1/3$ and of $\beta = 0.05$. The control parameter scenario $C(t)$ is chosen in this paper from the class schematically described in Fig. 1b. The scenario is thus markedly different from that studied in [12, 13] where forcing was switched off exponentially in time until no forcing was applied any more and a resting state was reached. With a constant driving amplitude $C_0 = 1.6$ or 2, the dynamics is chaotic and possesses an underlying chaotic attractor of large extension [35].

With any *constant* C, Eq. (S1) is invariant under the transformation $x \to -x$, $y \to -y$, $t \to t - \pi$ ($\pi = T/2$). As a consequence, any periodic attractor appears in equivalent pairs whose trajectories in the (x, y) state space are mirror images (with respect to the origin) of each other with respect to the origin. In other words, each clockwise rotating attractor has a counter-clockwise rotating counterpart. A particular example is provided by S. Fig. 1 presenting the two period-two limit cycles existing at a C value where no other attractors exist.

Heavy dots mark the position of the two-cycles on a stroboscopic map taken at $t = 0$ mod T, the map used throughout the paper. One sees that no symmetry remains, and this explains why the bifurcation diagram of Fig. 1a is not symmetric with respect to the $x = 0$ axis. Taking stroboscopic sections at other phases would lead to other pairs of dots on the map. The invariance property mentioned implies that with a phase difference of π, i.e. on stroboscopic sections taken at $t = T/2$ mod T, the period-two limit cycles are represented by colored circles which are mirror images of the ones of the other color on the $t = 0$ mod$T$ sections. Accordingly, the bifurcation diagram taken at $t = T/2$ mod T would be the mirror

image of Fig. 1a with respect to the $x = 0$ axis.

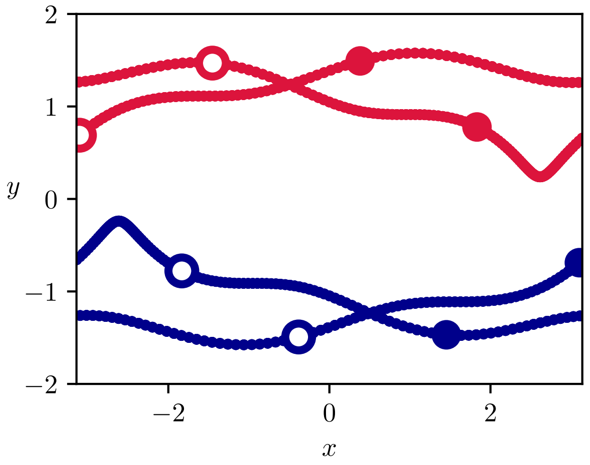

Figure 1: Time-continuous phase space trajectories of the clockwise (blue) and counter-clockwise rotating (red) attractors at C=0.5835. Attractors on the stroboscopic map taken at $t = 0$ mod$T$ are indicated by heavy dots. Colored circles indicate the attractors on sections taken at $t = T/2$ mod T.

These invariant properties do not hold, however, with time-dependent amplitudes $C(t)$. The clockwise and counterclockwise rotating snapshot attractors are then not equivalent, since the ramp starts at $t = 0$ (and not at $T/2$). An invariance property still exists: with any time-dependent $C(t)$, Eq. (S1) is invariant under the transformation $x \to -x$, $y \to -y$, $t \to t - \pi$ $(\pi = T/2)$ *and* $C(t) \to C(t - T/2)$. Any trajectory starting at x_0, y_0 with the scenario $C(t)$ is equivalent to the trajectory starting at $-x_0$, $-y_0$ with the scenario $C(t-T/2)$. The corresponding snapshot attractors should then be equivalent, too. Since in the bulk of the paper we do not shift the time in $C(t)$, attractors which are equivalent with constant C lose this property in the ramped system for $t > 0$.

SUPPLEMENTARY DISCUSSION II: ADDITIONAL SCENARIOS

In this section we also investigate scenarios differing from those discussed in the main text. S. Fig. 2 shows the new parameter intervals along the perimeter of the bifurcation diagram.

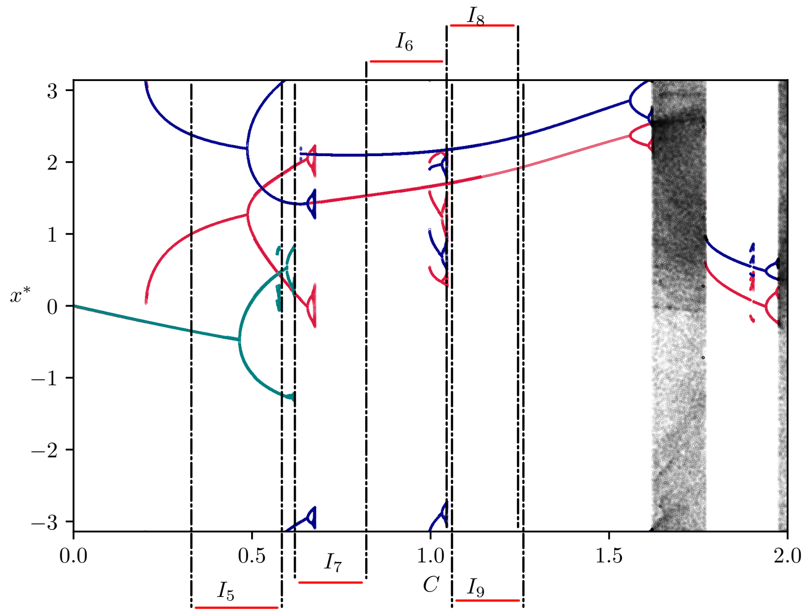

Figure 2: Bifurcation diagram as in Fig. 1a but with the control parameter intervals I_n, $n = 5, \ldots, 9$ investigated solely in the supplementary discussions, marked by line segments along the frame of the bifurcation diagram.

A. Dependence of the basins on t_r with control parameter interval I_2

In S. Fig. 3 we show the t_r-dependent basins for scenarios involving interval I_2 considered in Section 5.

In contrast to Fig. 2, the basins contain very fine filaments, even more pronounced when t_r is increased (corresponding to a less steep ramp). A filamentary, fractal structure is present everywhere (even for long observation times), and it deforms as t_r changes.

We have determined the tipping probabilities from the cyan attractor at C_-. Fig. 5a in Section 5 shows the dependence of these tipping probabilities on t_r. The functions presented indicate that even for relatively large values of t_r the tipping probabilities $P_{Cy,B}, P_{Cy,R}$ are not yet zero. The expected decay of the tipping probabilities to zero starts only at much larger values as discussed in Section 5.

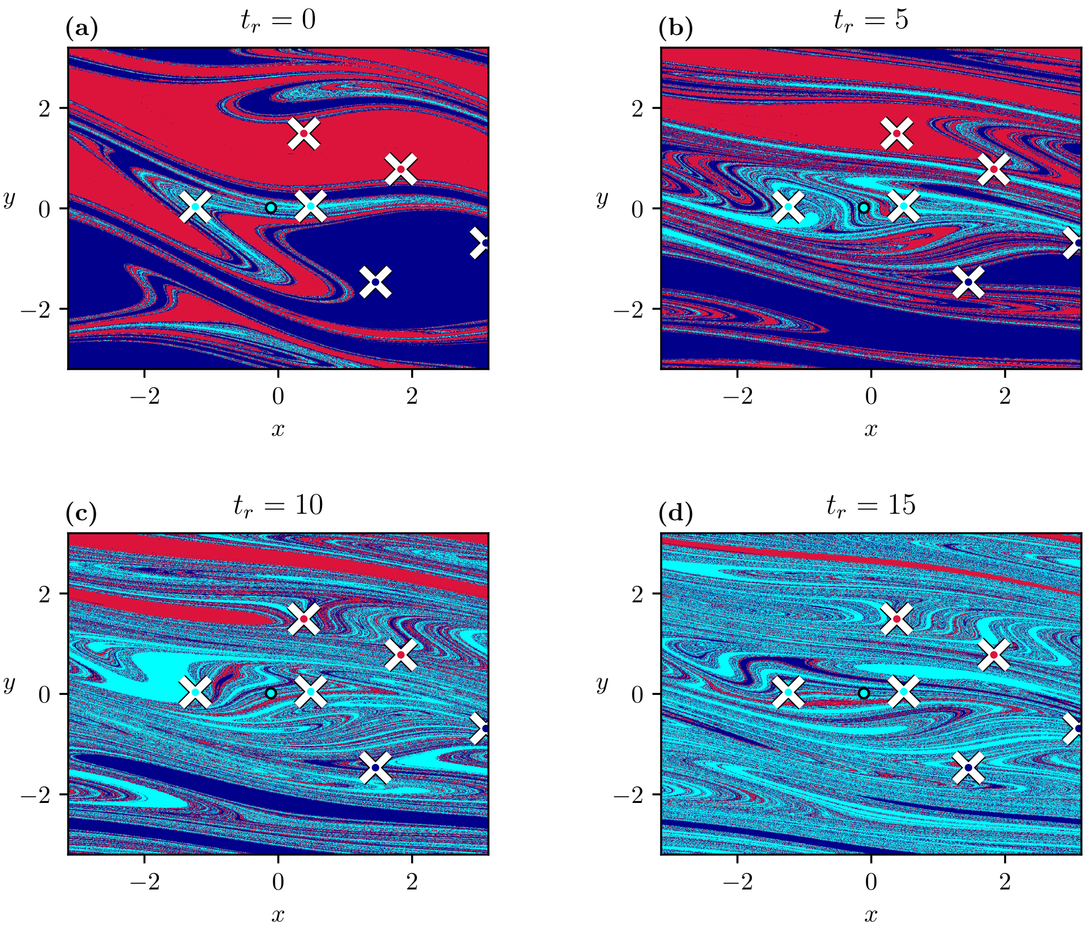

Figure 3: Basins of attraction for scenarios starting at $t_- = 0$ with $C_- = 0.1$ and ending at $t_+ = 100$ with $C_+ = 0.5835$ (I_2 in Fig. 2a). For each scenario, t_r is indicated above the panels. The black circle marks again the location of the cyan attractor at t_-.

B. Scenarios starting from a C_- with multiple coexisting attractors

As an illustrative example, we choose $C_- = 0.33$ (with three period-one attractors in the frozen-in system) and $C_+ = 0.5835$ (with three, period-two attractors), interval I_5. When calculating the tipping probabilities in this case, one should take into account in Eq. (3) that $B_1(t_-)$ is only a portion of the full phase space, since 3 attractors coexist in the initial state. To get these portions, first, one has to calculate the relative basins of attraction of A_1 at C_- and then to compute the tipping probabilities only for initial conditions starting in $B_1(t_-)$.

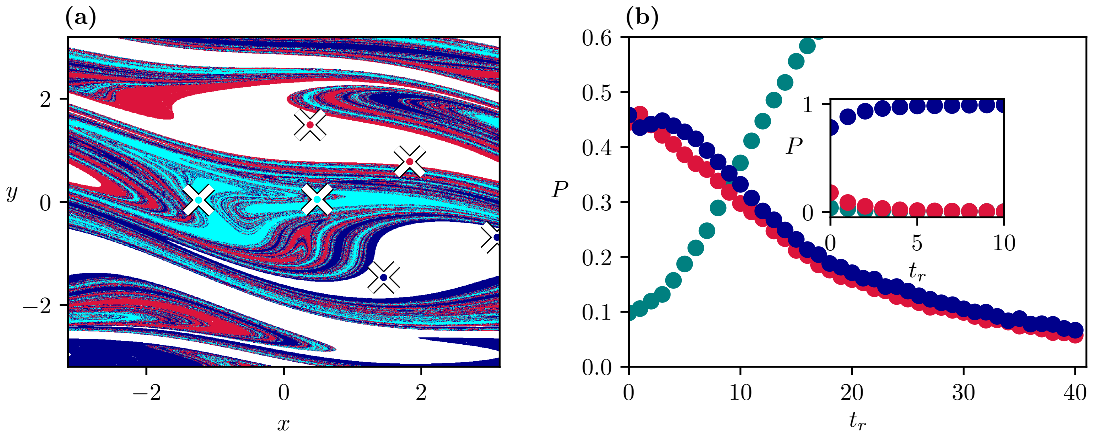

Figure 4: Scenario-dependent basins and tipping probabilities for $C_- = 0.33$, $C_+ = 0.5835$ (I_5), $t_- = 0$, $t_+ = 100$. (a): the basins are shown with $t_r = 8$ on the cyan attractor's basin of attraction at C_- and $t = 0$. (b): calculated tipping probabilities from the cyan attractor as a function of t_r. The tipping probabilities from the blue attractor are shown in the inset.

The scenario-dependent basins restricted to the basin of the cyan attractor at C_- are shown in S. Fig. 4a along with the tipping probabilities given in S. Fig. 4b. We note that the tipping probabilities disappear by about $t_r = 50$. Tipping probabilities can also be determined from any of the attractors at C_-. The inset shows these from the blue attractor (the results for the red one are very similar). One sees that a tipping into a different attractor is here also rather unlikely, and more or less the same for both the red and the blue attractor. The probabilities drop below 10^{-3} already when $t_r = 17$. From a practical point of view such small probabilities are unlikely to be measurable in real systems, therefore we claim that a rate-induced tipping is found here, but - as in earlier cases - the transition (at about $t_r = 17$) is not abrupt, but rather gradual and smooth as t_r increases.

C. Tipping into small-size chaotic attractors

Next we take a scenario (I_6) which ends at a value where chaotic attractors coexist with the two basic rotating attractors. Even if the chaotic attractors are small, an essential difference in comparison with previous cases of the Supplementary Discussion is that tipping

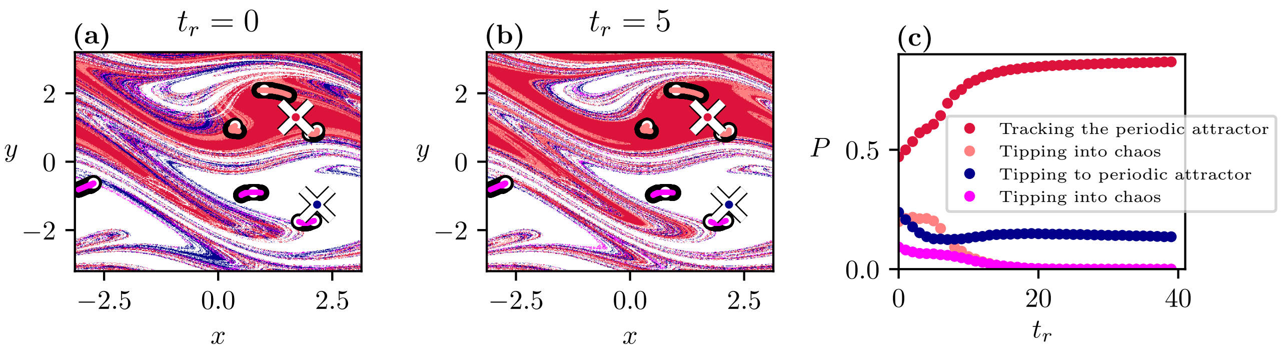

Figure 5: Scenario-dependent basins in a scenario starting with $C_- = 0.82$ and ending at $C_+ = 1.045$ (I_6) where two small 3-piece chaotic attractors coexist with the two basic overturning limit cycles, starting from the basin of attraction of the red limit cycle, $t_- = 0, t_+ = 100$. (a) [(b)]: $t_r = 0$, frozen-in [$t_r = 5$]. The chaotic attractor are encircled with a black line (c): Tipping probabilities into each of the attractors at C_+ as a function of t_r. Tipping probabilities from the chaotic attractors (orange and magenta) decrease with t_r and vanish at about $t_r = 15$.

might occur here into *intervals* not only into points in the stroboscopic map, or from a dynamical point of view, into unpredictable complex motion, not only into limit cycles. As in Section 6, we call this type of tipping *tipping into a chaotic attractor*. The colors used here to mark the basins of the chaotic attractors are lighter versions of the colors of the neighboring periodic attractors. The initial conditions are chosen from the interior of the basin of the red attractor at C_-. In the absence of a ramp, every point shown would fall onto the red attractor. In the presence of a ramp, other long-term outcomes are also possible.

The message of S. Fig. 5a,b is that the basin structure is more complex than previously. In S. Fig. 5c the tipping probability from the red limit cycle to the blue limit cycle and to the blue and red chaotic attractor is computed as a function of t_r, along with the tracking probability of the red periodic attractor. The probability of tipping to the chaotic attractor is nonzero only for t_r smaller than about 15, indicating that tipping into small-size chaotic attractors is rate-induced within the interval $t_r = [0, 40]$.

D. Comparison of scenarios with identical control parameter increases

We compare four scenarios having approximately the the same increase in the driving amplitude $\Delta C \approx 0.2$ started at different values of C_-. The scenario $C_- = 0.62, C_+ = 0.82$ (I_7) is to the left of the interval (I_6) investigated in the previous subsection, while scenario $C_- = 1.06, C_+ = 1.26$ (I_8), is to the right. In both cases the parameter drifts through small size chaotic attractors (see Fig. 2a). The last scenario $C_- = 1.045, C_+ = 1.245$ (I_9) corresponds to starting on a small scale chaotic attractor and ending on one of the rotating attractors (a case of tipping from a chaotic attractor, marked by symbols c). The tipping probabilities summarized in S. Fig. 6 are qualitatively similar, with a few differences. In the scenario with I_6, the tipping probability into the periodic attractors differs for $t_r < 15$ from the other cases since it is in this range where the probability of tipping into a (small scale) chaotic attractor is nonzero, see S. Fig. 5c. The tipping probabilities remain finite in all cases in the full t_r range investigated. The slight slopes remaining by $t_r \approx 40$ suggest a perpetual decrease of the blue curves. They are estimated to approach zero at a few hundreds of the period. This corresponds to the adiabatic limit, in which points started in the basin of the red attractor should remain in its slowly changing basin forever.

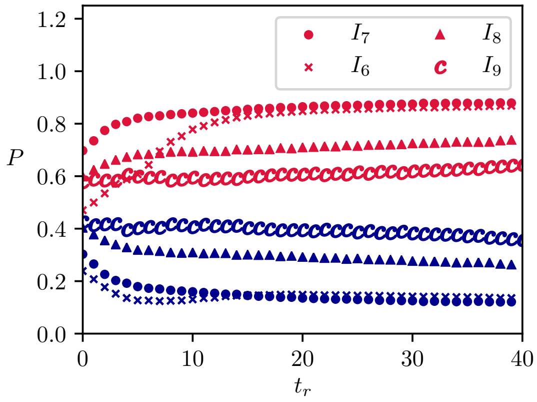

Figure 6: Tipping probabilities computed as a function of t_r for four scenarios marked by different symbols. The red (blue) curves indicate the probability of tipping into the red (blue) limit cycle at C_+ from the basin of attraction of the red limit cycle at C_-. ($t_- = 0$, $t_+ = 100$.) The graphs marked by symbol c represent the tipping probabilities from chaos into one of the periodic attractors.

SUPPLEMENTARY DISCUSSION III: BIVARIATE TIPPING PROBABILITIES

As described in the main text, the tipping probabilities might exhibit a complicated dependence on both the ramping time t_r and the starting time t_-, for a fixed choice of C_- and C_+. Here, these bivariate functions are shown for the three types of transition that exist in the range I_2. For completeness, the second row shows these probabilities color coded on the plane t_r,t_-. Red lines mark the slices shown in Fig. 5.

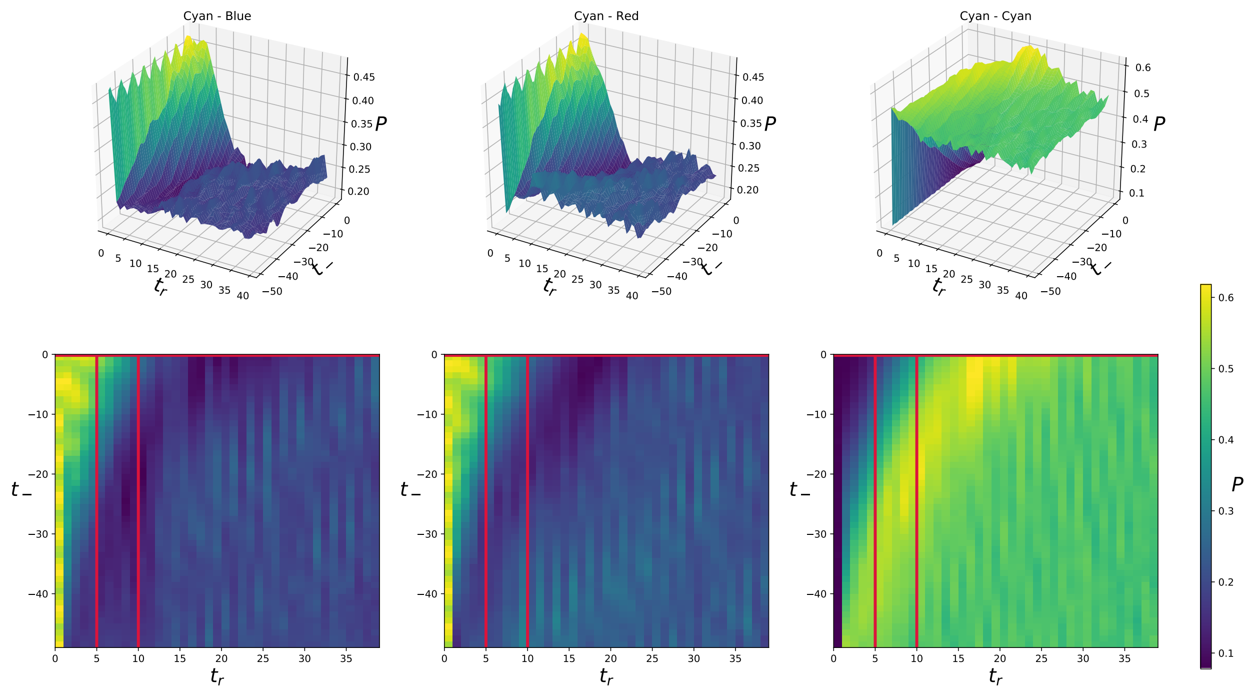

Figure 7: Tipping probabilities measured in scenarios with $C_- = -0.1$ and $C_+ = 0.5835$ (I_2). Here $P_{Cy,B}$, $P_{Cy,R}$ and $P_{Cy,Cy}$ are displayed in the three columns as bivariate functions of t_r and t_-. The probability is represented as a variable along the vertical axis in the first row, and as a projection, color coded, in the second row.

SUPPLEMENTARY DISCUSSION IV: SNAPSHOT ATTRACTORS AND THEIR ROLE IN TIPPING PHENOMENA

In order to discuss the role of snapshot attractors in the tipping transitions, we consider two scenarios, summarized schematically in S. Fig. 8. These differ only in their initial time

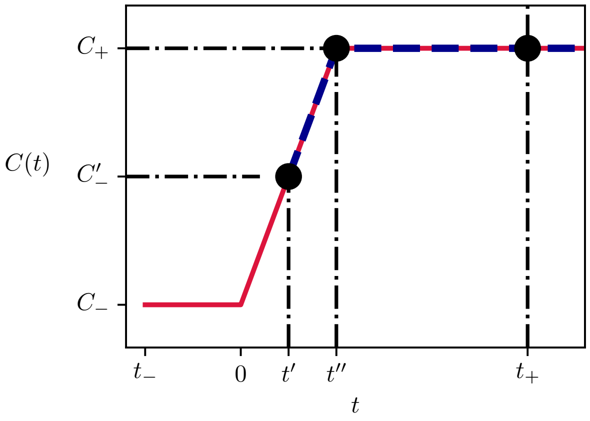

Figure 8: Schematic diagram of scenarios A: (C_-, t_-, C_+, t_+, r), red line, and B: $(C'_-, t'_- = t', C_+, t_+, r)$, blue dashed line, with the same ramp r, (see Eq. (1)), whose basins of attraction are compared in S. Fig. 9, and during which snapshot attractors are presented in S. Fig. 10.

(or initial parameter values C_- and C'_-) while having the *same ramp* and identical futures. The earlier initialization time shall be taken as $t_- = -4$ on the initial plateau, and the later initialization time is on the ramp at $t' = 2$. The general parameters of these scenarios are:

- A: (C_-, t_-, C_+, t_+, r),
- B: $(C'_-, t'_- = t', C_+, t_+, r)$.

The initial control parameter C_- is taken to be 0.1 with the cyan attractor and C'_- is chosen (for convenience) to be such that the cyan attractor is also the only stable one at this larger value. The endpoint is selected to belong to three coexisting periodic attractors, with $C_+ = 0.5835$, which has been taken in the main text, too (see e.g. Fig. 6). The novelty of this choice is the ability to compare scenarios which start on the initial plateau and on the ramp, but are otherwise the same.

The scenario-dependent basins of attraction (A and B) are remarkably different, as illustrated by S. Fig. 9a-b. We have also determined the tipping probabilities $P_{Cy,R}$ and $P_{Cy,B}$ which are the only relevant ones in both scenarios, and the results are summarized in S. Table I. The difference can be understood intuitively by looking at the patterns of S. Fig. 9. The total area of the basins, colored red and blue are approximately identical, and cyan

dominates them in (a) ($P_{Cy,Cy} = 0.558$), while red and blue are somewhat more prominent than cyan in (b) ($P_{Cy,Cy} = 0.264$).

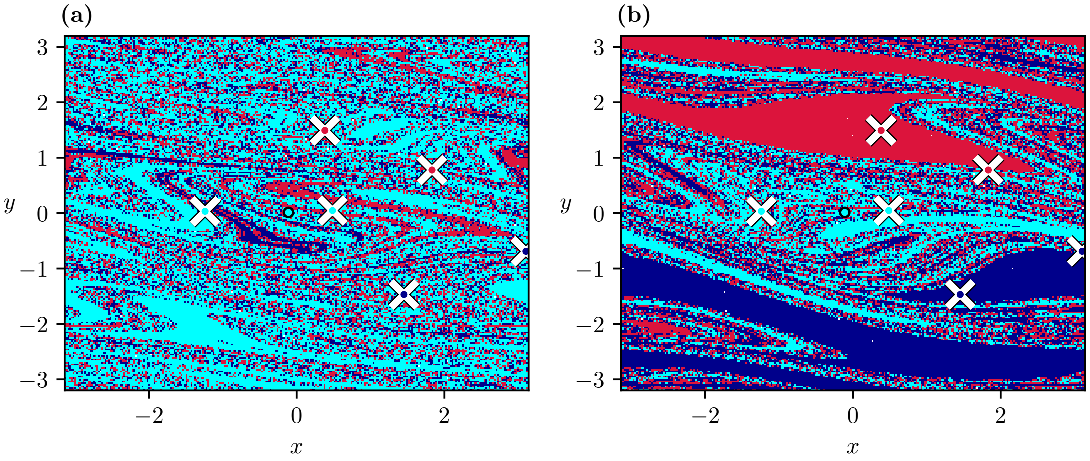

Figure 9: Scenario-dependent basins of attraction for scenario A (a), and for scenario B (b).

	$P_{Cy,R}$	$P_{Cy,B}$
Scenario A	0.237	0.205
Scenario B	0.355	0.381

Table I: Tipping probabilities $P_{Cy,R}$ and $P_{Cy,B}$ computed for scenarios A and B. The tracking probability is $P_{Cy,Cy} = 1 - P_{Cy,R} - P_{Cy,B}$.

The explanation of the differences of the two panels of S. Fig. 9 (and the two rows of S. Table I) lies in the difference between the initial condition for the continuation of scenario A, at the instant $t' = 2$ when the value $C'_- = C_- + rt' = 0.1967$ is reached along the ramp, and the uniform initial distribution applied as an initial condition to scenario B at the same time instant. To this end, we make use of the property that the ramped system is memory free (see Eq. (S1)). As a consequence, any instantaneous state of the ensemble provides the initial condition for its future. The initial condition for the continuation of scenario A at t' is nothing but the distribution evolved from the uniform initial distribution over the full state space applied at time $t_- = -4$. Since the convergence time to a snapshot attractor is about 3 time units in this system as shown in [12], and $t' + |t_-|$ is beyond this value, we can consider

this initial condition at t' to be the snapshot attractor along with its natural distribution belonging to this time instant in scenario A (started at $t_- = -4$). This snapshot attractor is shown in S. Fig. 10a. The initial condition for scenario B starting at $t'_- = t'$ and C'_- is the full phase space, it is thus the difference between a uniformly filled out rectangle and a much "smaller" filamentary pattern with a nontrivial distribution, the snapshot attractor of S. Fig. 10a, which is the cause of the differences in the tipping transitions.

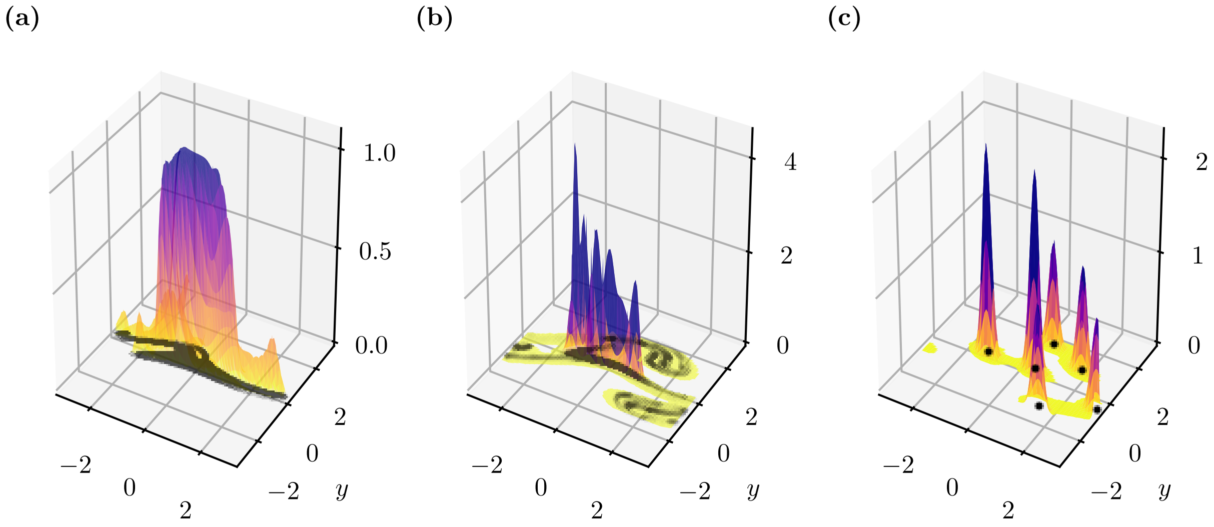

pine

Figure 10: Snapshot attractors with their distributions belonging to the black dots in S. Fig. 8 along scenario A with a rate $r = 0.04835$, $C_- = 0.1$, $C_+ = 0.5835$, $t_- = -4$, $t_+ = 100$. (a): snapshot attractor evolved from the uniform initial condition taken at $t_- = -4$ by time $t' = 2$, (b): by time $t'' = 10$. (c): snapshot attractor taken at $t_+ = 100$ which exhibits 3 pairs of peaks designating the 3 period-2 attractors on the C_+ plateau. Note that the highest pairs of peaks belongs to the cyan attractor (see S. Table I).

For completeness, in S. Fig. 10b we show the snapshot attractor evolved in scenario A by time $t'' = 10$ when the plateau of $C_+ = C_- + rt''$ is reached. This can also be considered as the image of the object visible in S. Fig. 10a with respect to the ramped dynamics.

In addition, it is to be noted that the shape and distribution of the snapshot attractor at t' and t'' is *independent* of the future of the scenario. They are e.g. the same in any scenario, which is identical to A up to t'' and only differing in their end values and plateaus.

The similarity of these snapshot attractors and their distribution to usual chaotic attractors and their natural distribution (see e.g. the cover plate of [35]) might be surprising.

The explanation of this similarity lies in the fact that transient chaos is ubiquitous for any frozen-in case for $C > C_b = 0.2$, and it is the filamentary, fractal *unstable manifold of the chaotic saddles* that determines the shape of the snapshot attractors in the ramped system, as pointed out in [12, 13].

The snapshot attractor into which the object shown in S. Fig. 10b evolves by time t_+ along the scenario A, is shown in S. Fig. 10c. Its particular form is due to the fact that the system left the ramp by t'' and has been evolving according to the frozen-in dynamics with C_+ on the plateau since then. The six sharp peaks correspond to the three period-2 limit cycles representing the asymptotic attractors of the frozen-in system. In this view, the tipping probabilities, in scenario A, from the cyan attractor at $C_- = 0.1$ into any of the limit cycles appear to be proportional to the strength of the corresponding pairs of peaks of the snapshot attractor at t_+.

In summary, the difference in the tipping probabilities of scenarios A and B is caused by the difference of the distributions at C'_-, t', which is a uniform one in scenario B. This difference is reflected in the different heights of the peaks on the periodic attractors belonging to the final time t_+. Consequently, the periodic attractors belonging to scenario B at C_+, t_+ consists of 3 pairs of peaks but of strengths differing from those of S. Fig. 10c, in harmony with the difference of the tipping probabilities. In particular, as the second row of S. Table I and S. Fig. 9b suggests all 3 pairs should be of similar height in this case.
